# Grading Pseudofractures—The “Breach–Beak–Bump–Bridge” Approach

**DOI:** 10.1007/s00223-025-01371-z

**Published:** 2025-04-16

**Authors:** Lothar Seefried, Dominik Rak, Franca Genest

**Affiliations:** Department of Osteology and Clinical Trial Unit, Orthopedic Hospital, König-Ludwig Haus, Julius-Maximilians-Universität Würzburg, Brettreichstraße 11, 97074 Würzburg, Bavaria Germany

**Keywords:** Pseudofracture, Hypophosphatasia, 4B scale, Osteomalacia

## Abstract

Pseudofractures are atraumatic radiolucencies resulting from compromised bone mineralization and are often associated with poor clinical outcomes in patients with skeletal disorders. The incidence, clinical course of healing, and the risk of recurrence of pseudofractures are not well characterized, not least because pseudofractures and fractures are regularly reported under the general term “fractures,” despite underlying pathophysiological differences. Accordingly, this report is intended to conceptualize a grading scale for identifying and assessing pseudofractures. The scale was developed based on our clinical experience with. The proposed taxonomy includes 4 radiographically distinct stages, progressing from an unreactive initial Breach (Stage 1) to a stage with a visible Beak (Stage 2), appearance of a rounded Bump (Stage 3), and formation of a Bridge (Stage 4) across the interline. These scores correspond to radiographic transformations observed along the course of pseudofracture consolidation, although these stages of healing are reversible, and stagnation or relapse may occur at any stage. Dislocation should be indicated by adding a “d” to the score; adding an “s” indicates that the bone is clinically stable, meaning pain-free full weight-bearing is possible, because of surgical stabilization or sustainable cortical bridging (typically in Stage 4 or 0 [consolidation]). The scale may be used for any pseudofracture regardless of anatomical site or etiology. The proposed Breach–Beak–Bump–Bridge (4B) concept is a tool that can be used in clinical practice to assess pseudofractures over time and to improve specificity and clarity in communication of these findings.

## Introduction

Pseudofractures are atraumatic radiolucencies that typically occur perpendicular to the longitudinal cortical axis of a bone, most commonly—but not exclusively—along the diaphyseal segment of tubular bones [1, 2]. Contrary to what the name suggests, pseudofractures (i.e., Milkman lines or Looser zones) are discontinuities detected radiographically that do not result from causative trauma but from focally compromised bone mineralization and/or remodeling [1, 2]. These pseudofractures typically do not span the diameter of the bone but rather affect only part of the cortex, as seen on plain radiographic films. Because the process is essentially reversible and may be associated with a physiologic healing response emerging from periosteal or endosteal tissues, lesions may show subtle callus formation and scleroses at the margins [1, 2]. Pseudofractures can usually also be distinguished from common, traumatic fractures by their differential localization and morphology [2].

Although sometimes referred to as insufficiency fractures, pseudofractures have a distinct pathophysiology. While insufficiency fractures typically result from normal stresses acting on compromised bone with insufficient resilience [2, 3], mechanical stress or trauma is seemingly not essential for the occurrence of pseudofractures, even though they frequently become symptomatic after an impact triggers pain [3]. Pseudofractures are a focal radiographic equivalent of compromised mineralization and regeneration [2] but may become clinically evident with prodromal pain and eventually appear radiographically as a complete discontinuity/fracture after a minor trauma [2].

Pseudofractures are indicative of compromised mineralization (i.e., osteomalacia) that can result from any one of multiple conditions, most frequently but not exclusively systemic or focal imbalances in calcium or phosphate homeostasis [1, 4]. Well-established, potential causes of compromised mineralization include long-standing vitamin D deficiency, phosphate-wasting disorders, and deterioration of cells or enzymes regulating mineralization, such as what occurs in hypophosphatasia (HPP) or long-term antiresorptive treatment with bisphosphonates (i.e., pyrophosphate analogs) [1, 5–8]. Unfortunately, data on the actual incidence of pseudofractures in association with specific disorders, including their clinical course of healing and recurrence, are sparse. One major cause for this dearth of clinical understanding comes from the fact that pseudofractures and fractures are typically reported in common under the umbrella term of “fractures.” Further, there is no taxonomy to score incident pseudofractures that takes into consideration their progression or level of remineralization progression. However, it is important to understand and specifically document these different manifestations, since correct identification would guide the clinician to intervene accordingly in a timely manner.

Pseudofractures are most frequently observed in weight-bearing lower extremities, mainly the femur and metatarsals [2], which may suggest a mechanical component in the pathophysiology. However, such fractures have also been observed in other bones [2, 9], and it is very possible that focal demineralization in non-weight-bearing or less weight-bearing parts of the skeleton goes unnoticed because it does not become symptomatic. Either way, most of what is known about pseudofractures comes specifically from the femoral site. Reflecting their pathophysiology, it is not surprising that these discontinuities exhibit poor healing response with cortical beaking or flaring due to altered mineralization and turnover [2]. Pseudofractures can eventually consolidate and heal, but some patients experience long-term persistence, recurrence, or progression of these lesions and, in some instances, complete fractures after minimal trauma [2].

Two well-studied conditions regularly associated with a high prevalence of pseudofracture are the hereditary disorders HPP and X-linked hypophosphatemia [10, 11]. Among adult patients from the Global HPP Registry prior to treatment with the enzyme replacement therapy asfotase alfa, 53.5% were reported to have recurrent or poorly healing fractures/pseudofractures [10]. In a study of burosumab in patients with X-linked hypophosphatemia, 29% (4/14) had pseudofractures prior to treatment initiation [11]. In another study of burosumab in patients with X-linked hypophosphatemia, unfortunately and in line with the above, pseudofractures and fractures were aggregated, despite their different etiology [12]. However, from the clinical outcomes of treatment in these patients, we have learned that targeted treatment to restore mineral homeostasis and mineralization can facilitate healing of these lesions [11, 13].

Protracted pseudofractures and healing complications are common, especially if the underlying metabolic condition cannot be amended substantially, and some fractures persist for many years. As a result, the burden of disease in patients, for example, in those with HPP, can be substantial and result in considerable disability, pain, and reduced quality of life [14, 15]. Delays in recognizing and diagnosing pseudofractures are common and can worsen the situation over time as the lesions and the associated instability progress [16]. Indeed, in advanced situations, specifically when the underlying mineral disorder cannot be corrected, patients require surgical stabilization of the bone. Considering prolonged healing periods, the latter should be accomplished preferentially with sustainable, intramedullary, load-sharing devices at low risk for material fatigue [17].

## Assessment of Fracture Healing

There are no established prognostic criteria or metrics, nor formal treatment guidelines for diagnosing or determining the severity, prognosis, or management of a pseudofracture [2]. Consequently, treatment decisions regarding surgical or conservative treatment are based on subjective individual assessment. Furthermore, monitoring the success of treatment is also limited because assessments extrapolated from osteoporosis and other disorders do not appropriately reflect the pathology and course of remineralization. Adequate assessment of the state of deterioration or healing is critical for ensuring proper treatment strategies in terms in invasiveness and timing of weight-bearing activity [18]. While the Radiographic Union Score for Tibial Fractures (RUST) and its modifications, such as the modified RUST (mRUST) and the Radiographic Union Score for Hip (RUSH), are useful in assessing healing of tibial or femoral fractures after nailing and healing of hip fractures [19, 20], these tools are not suitable to assess the progression and healing of pseudofractures.

Based on our experience with caring for patients prone to develop femoral pseudofractures (HPP > 500 / > 100 compound heterozygous; XLH > 100, OI > 100), we propose a grading system for characterizing femoral pseudofractures and documenting the radiographic appearance of pseudofracture in these conditions. The proposed Breach–Beak–Bump–Bridge (4B) Scale provides a model for assessing and managing pseudofractures regardless of their etiology.

## 4 Bs to Bone Healing

The proposed 4B Scale is based on the natural course of changes seen on plain radiographs and, for consistency, first focuses on femoral fractures since the femur appears to be the best-evaluated anatomical site of pseudofractures, especially in patients with HPP [2]. However, similar traits along the course of remineralization are also seen in pseudofractures at other skeletal sites [2]. Details of the proposed scale and representative illustrations and radiographs depicting each stage of healing progression are presented in Table 1.

**Table 1:**
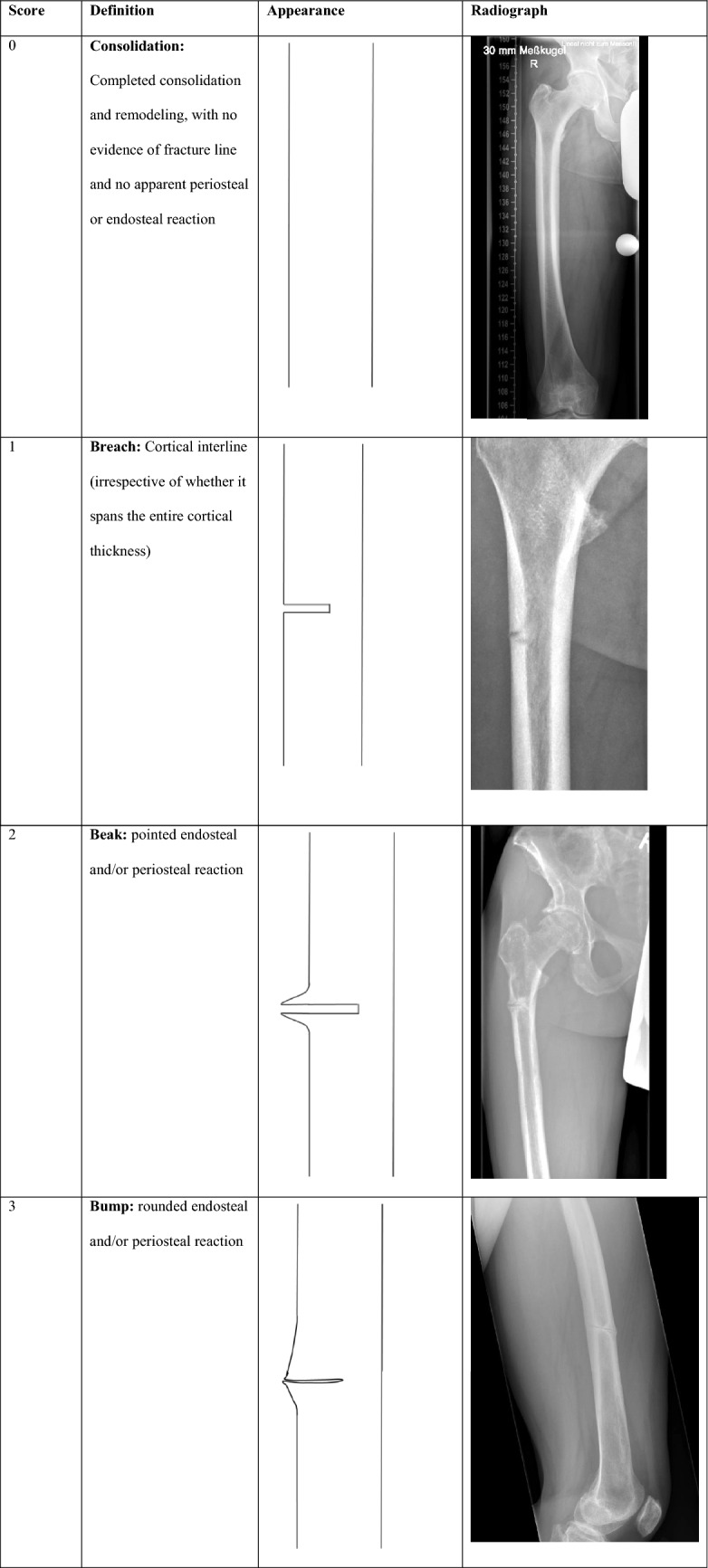

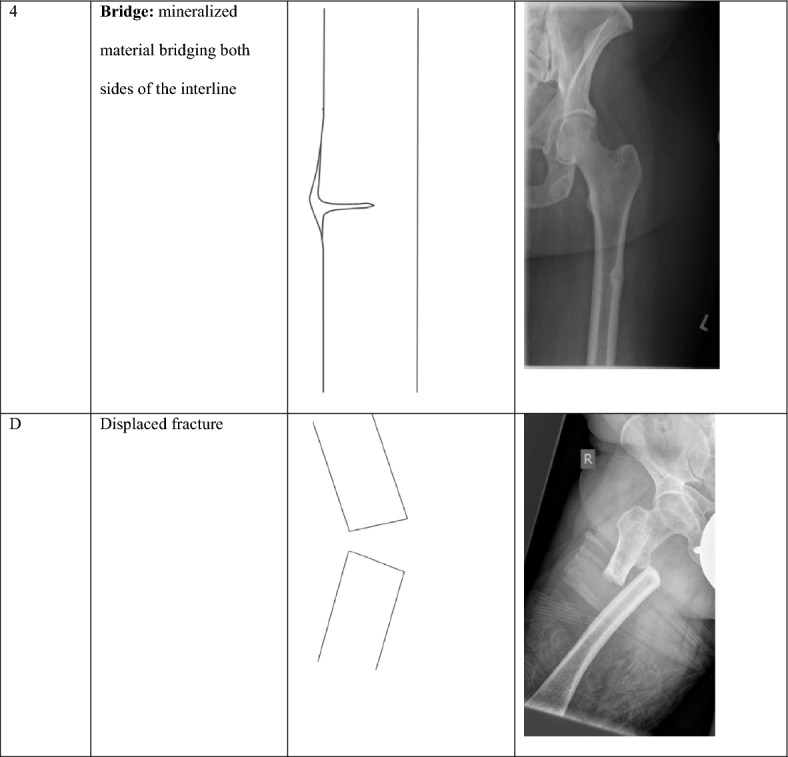
Breach–Beak–Bump–Bridge: the 4B Scale

As a general rule, and for clinical applicability, assessments for the 4B Scale are always based on the single radiograph showing the worst (i.e., most severe) appearance when standard X-ray films in two or more planes are available. A simple summary of the underlying observation is that pseudofractures undergo a typical sequence of transformations from a Breach to the development of one or more sharp Beaks at the margins of the interline, followed by transformation to a rounded Bump, with mineralized material eventually bridging across the interline. With progressive mineralization, this bridge eventually becomes mechanically stable and load bearing. Although there is no defined threshold to assign stability to bridging tissue, an intuitive and experience-based suggestion is to assume stable bridging when the diameter of mineralized tissue along the Bridge equals the thickness of the unaffected cortex nearby and weight bearing is free of pain.

Specifically, a score of 1 represents a blank, nonreactive interline (Breach) that may or may not span the entire cortical width on a particular image. In our experience, there is no clinically relevant difference between a pseudofracture that does and one that does not encompass the entire cortical width. Once the breach is visible, demineralization and associated mechanical compromise can be confirmed; however, there is a need to differentiate fractures that have progressed to complete discontinuity with consecutive dislocation of the two bone fragments, typically following some minor trauma. Such dislocation may occur at any stage and is indicated by adding a “d” to the respective score (i.e., 1d, 2d, and so on). Because discontinuity implicates disruption of any connecting organic tissue including unmineralized tissue, mere reconstitution of mineralization is insufficient to restore cortical integrity and stability, open reduction, and internal fixation typically are required to attain mechanical stability and enable osseous healing.

Progressive levels of healing after a Breach (score of 1) are indicated by evolution of a (sub-)periosteal edgy Beak (score of 2) reflecting initial mechanisms to restore stability. Still, if compromised mineralization persists, this rarely progresses to complete consolidation. More frequently, continued remodeling and adaptations of mineralized paracortical tissue may lead to formation of a rounded Bump (score of 3). However, it remains to be elucidated if transitions from 2 to 3 reflect a physiological continuum or if these represent distinct response mechanisms depending on individual aspects of mineral homeostasis and focal micromobility. Progressive mineralization may eventually form a Bridge across the interline (score of 4). Once a bridge has formed and consolidates further, it ultimately constitutes a stable, load-bearing bridge. Clinical stability, however, cannot be confirmed by radiographs alone in this setting of very subtle findings and, for communication purposes, we suggest adding an “s” when radiographic bridging is associated with clinical stability (i.e., Grade 4 s) or if the situation is stabilized, e.g., with a surgical implant despite persistence of the interline w/o Beak/Bump (i.e., Grade 0 s/1 s/2 s/3 s).

**Fig. 1:**
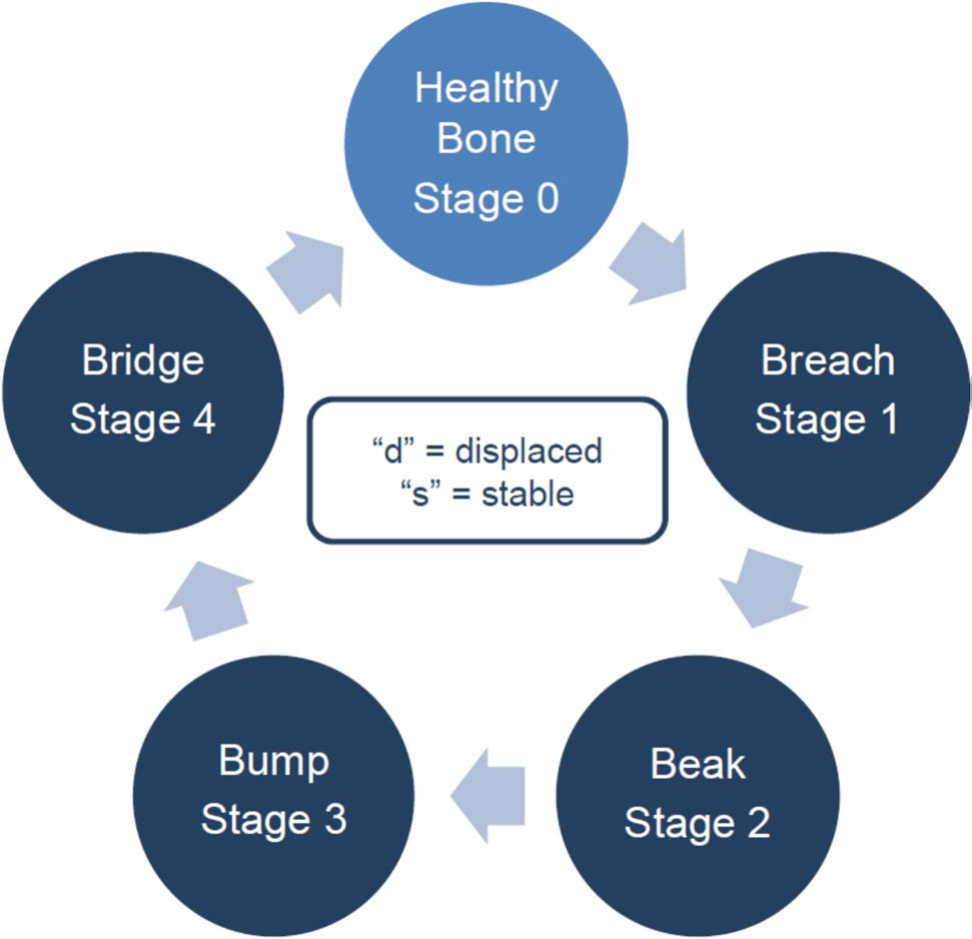
Visual representation highlighting the dynamic nature of the “Breach—Beak—Bump—Bridge” approach

Since progression and regression of pseudofractures constitute a perpetual issue in mineralization disorders, this has to be regarded as a cyclic process and a radiographic Stage 0 is included to indicate the absence of any signs of a pseudofracture, either because radiographic signs are not yet apparent or because bone around a preexisting pseudofracture has remodeled completely (Fig. 1). In both instances, this does not exclude radiographic signs from becoming apparent over time. In this regard, the 4B Scale does not indicate a straightforward, irreversible progression of healing and consolidation and explicitly includes the potential for relapse. Recurrence of a pseudofracture is possible at any stage; in most cases, recurrence from an advanced stage of healing involves redevelopment of a visible interline.

Importantly, while periosteal bridging is a critical requirement for finally developing a sustainable structure, it is critical to always include the clinical perspective when defining a stage of healing progression and to make sure a radiographically visible bony bridge is sustainable and capable of load bearing. This is especially true since it is not uncommon that, particularly in patients who are diagnosed late, the interline may be too faint to be visible on a radiograph. In this situation, presence of a cortical Beak or Bump in combination with focal pain has to be considered an indirect indicator of a prevalent pseudofracture which should be assessed as Grade 2 (sharp/edgy Beak) or Grade 3 (rounded Bump), accordingly.

As mentioned above, pseudofracture healing can be a protracted process, with any stage persisting for a substantial period of time and recurrence possible at any stage. Therefore, healing is not finished until complete remodeling and cortical realignment have been reached (i.e., no visible sign of interline and no apparent periosteal or endosteal reaction, equivalent to a score of 0). However, even then recurrence can occur, specifically when mineral homeostasis and bone remodeling become unbalanced again.

While the 4B stages “Breach—Beak—Bump—Bridge” are intended for assessing, staging, and monitoring the mineralization course of pseudofractures, addition of the letter “s” for a stable situation or “d” for displaced fractures supports clinical utility of the scale. For example, a clinically suspected pseudofracture precluding weight-bearing despite (as yet) unsuspicious X-ray can be assigned Stage 0, whereas a pseudofracture Stage 1 may be termed 1 s after surgical stabilization though it remains obvious that the mineralization process itself has not yet improved.

## Discussion

Differentiating pseudofractures from fractures is the key to their appropriate diagnosis and management. Considering the pathophysiology of these lesions with compromised bone metabolism and the absence of a causative trauma, it is obvious that the consolidation and healing of pseudofractures do not follow established mechanisms of primary or secondary fracture healing. As a consequence, pseudofractures are frequently associated with poor clinical outcomes in patients with skeletal disorders, especially when the pseudofractures are misdiagnosed as another type of fracture. Conversely, treatments that correct the underlying biochemistry and facilitate physiologic mineralization such as asfotase alfa in adults with HPP [13, 21, 22], play an important role in promoting pseudofracture healing. Therefore, approaches are needed to both appropriately diagnose these fractures and to also monitor their clinical course and response to treatment.

Although the proposed 4B Scale is based on individual experiences, it should be considered a starting point for a more specific diagnosis, documentation, and management of pseudofractures, regardless of the underlying condition. However, validation of the scale will be required for use in individual disorders, such as HPP, XLH, or osteogenesis imperfecta. In that regard, the 4B Scale is intended as an additional diagnostic tool complementary to well-established clinical practice. Further evaluation and potential refinement will also help in using this scale for future applications in terms of defining the severity and effect of medical interventions on the healing of pseudofractures.

The fact that the proposed scale builds upon the image with the most severe representation should not waive the general recommendation for conducting conventional radiographs in two planes. Sclerosis may also be present in a pseudofracture, suggesting unstructured mineralization after failed consolidation. The 4B Scale can be used to assess the stage of femoral pseudofractures both at diagnosis and for monitoring improvements during treatment. Notably, this scale is intended as an assessment of an individual pseudofracture in a specific cortex and not as an indicator of a patient’s overall clinical status when more than one pseudofracture is present.

A limitation of the proposed 4B Scale is that, for the time being, it is solely based on our clinical experience; however, there is no robust literature or compelling evidence from which to draw meaningful conclusions, as current reports lack the granularity the proposed scale intends to provide. As a result, the 4B Scale can be considered a starting point and subject to further refinement as we gain further knowledge. To keep it practical, the Scale intentionally refrains from taking into account any potential mechanistic factors, including deformities or potential traumatic impacts. Even if these factors interfere with the course of healing and the risk of recurrence, they do not fundamentally change the resulting radiographic appearance or the stages of healing.

## Conclusions

The 4B Scale was developed to enable a more nuanced clinical picture for diagnosing, documenting, and monitoring progress of healing of femoral pseudofractures for both clinical and scientific purposes. This Scale may help to standardize how the status and changes of pseudofractures are being assessed and discussed in the medical community to allow a more detailed perspective and harmonize management approaches. Eventually this Scale may also help identify and characterize patients who are most likely to respond to specific treatments.

## Data Availability

Not applicable for this manuscript, which provides the intellectual framework of a new grading scale based on our clinical experience.
